# Hospital Finance

**Published:** 1920-06-19

**Authors:** 


### HOSPITAL FINANCE.

My congratulations to the Provincial Hospital Secretary whose article bearing this title appeared in last week's The Hospital. If it possesses any fault, it is that it contains too much for one meal. The writer is an enthusiast and a specialist.

"The advertising of hospitals' needs," he says, "has in the past been on too stereotyped a basis." I pick this, haphazard, out of the condensed mass. In other words, hospital advertising has been in the hands of incompetents. Advertising is a high art, and it is much to be regretted that the people who most highly appreciate this fact are professional beggars. The honest man with sound merchandise to sell is too apt to think that the goods speak for themselves, which would be true were it not that the charlatan displays articles which look better and which cost less. The mass of the people are busy with their own concerns, and in addition they are unimaginative. I presume that the stereotyped method referred to by the writer of the article is the printing and publishing of hospital annual reports. A great many thousands of pounds are spent on these documents, and it is extremely doubtful if anybody outside the select members of any hospital staff ever reads one of them through. Tradition demands their publication, but it is a mistake to suppose that they are a means of publicity. The champions of the voluntary system are faced with the fact that the public, knowing in a general way of their existence and of their work, preserve with regard to them a detached mind. They feel no sense of responsibility or ownership, and hence when they have dropped their half-crown on the collection-plate on Hospital Sunday they experience a feeling of satisfaction, not to say gratification, that they have done their duty and a little more. The hospital report, in the waste-paper basket, and the parson's annual sermon, include the major part of hospital publicity effort. It is really a matter for surprise, in the circumstances, that hospital finance is not in even worse plight.

I make no apology for ringing the changes on this admirable article. The hour has struck for making a valiant effort to save the voluntary system from wreck. No spasmodic outcry can avail. The people must be trained to give now, and to keep on giving. It must be brought home to their consciences that sick-poor hospitals are their direct and intimate concern, and that within their walls the skilled physician and surgeon, as well as the trained nurse, learn their business. To teach the lesson effectually capable publicists must be employed, and the directors must be men and women of high intelligence and broad views.

Wherein consists publicity? The first essential to realise is that publicity does not necessarily consist merely of newspaper articles and popular lectures, valuable as these may be. It is a subject of common complaint at present that industrial workers derive great benefits from hospitals, that they are well paid, and that their contributions towards their upkeep are inconsiderable. I wonder if it has ever occurred to any of those responsible for hospital maintenance that the hospitals themselves, and not the workers, are responsible for this condition of things? Their detachment is not of their own making. It has been imposed upon them by the institutions concerned, and one of the most pressing necessities is the immediate reform of governing Boards. If the worker's sympathy and help are to be ensured, he must be given a share in the administration. However high-minded and worthy the members of Hospital Boards may be individually, collectively the Boards are not, in the true sense of the word, "popular." It is in view of such reform being effected that I suggest the necessity for men and women of high intelligence and broad view directing a hospital publicity department. The personnel of the governing body is in itself a vital factor of effectual propaganda. Taxation and representation is an old and a sound economic dogma. Together they form an integral part of our scheme of civilisation. Even now it is on the taxation and representation principle that Hospital Boards are formed. The governors are the givers. If the giving public is to be extended, the administration must be made to correspond.

### Hospital Workers.

Hospital governing bodies have as a rule proceeded on altogether wrong lines with regard to their own employés. Is it true or not that in making their "stereotyped" appeals for support the claims of nurses to just economic treatment have been altogether omitted? I have never seen them included. I see that Lord Knutsford wants £1,252 to pay last month's milk bill for the "London." A very proper cause for appeal! But all the appeals that I have ever come across are of this kind, and none is ever made for the nurse. When all the bills are paid she is the residuary legatee, and hence her hours remain too long and her wages too small. This does not make for the popularity of the voluntary system, and would militate against the success of a publicity campaign.

The subject is one of wide dimensions. Reform must be far-reaching and thorough. The house of the voluntary system must be set in order, otherwise the evil day will only be postponed for a little time, and the State will be forced to intervene, although it cannot be denied that the voluntary offerings of a nation on behalf of the sick and suffering are, in essence, the outcome of true religion. When for this is substituted the levy of a half-penny in the pound on the rates, we shall have automatically descended to a lower plane.

